# MiR-221/222 promote epithelial-mesenchymal transition by targeting Notch3 in breast cancer cell lines

**DOI:** 10.1038/s41523-018-0073-7

**Published:** 2018-08-06

**Authors:** Yuan-Ke Liang, Hao-Yu Lin, Xiao-Wei Dou, Min Chen, Xiao-Long Wei, Yong-Qu Zhang, Yang Wu, Chun-Fa Chen, Jing-Wen Bai, Ying-Sheng Xiao, Yu-Zhu Qi, Frank A. E. Kruyt, Guo-Jun Zhang

**Affiliations:** 1 The Breast Center, Cancer Hospital of Shantou University Medical College, 7 Raoping Road, 515031 Shantou, China; 2 ChangJiang Scholar’s Laboratory, Shantou University Medical College, 22 Xinling Road, 515041 Shantou, China; 3 Department of Medical Oncology, University of Groningen, University Medical Center Groningen, Hanzeplein 1, 9713 GZ Groningen, The Netherlands; 4 Department of Breast and Thyroid Surgery, The First Affiliated Hospital of Shantou University Medical College (SUMC), 57 Chang ping Road, 515041 Shantou, China; 5 Department of Pathology, The Cancer Hospital of Shantou University Medical College (SUMC), 7 Raoping Road, 515031 Shantou, China; 6 Xiang’an Hospital of Xiamen University, 2000 East Xiang’an Rd., Xiamen, China

## Abstract

Basal-like breast cancer (BLBC) is an aggressive subtype with a strong tendency to metastasize. Due to the lack of effective chemotherapy, BLBC has a poor prognosis compared with luminal subtype breast cancer. MicroRNA-221 and -222 (miR-221/222) are overexpressed in BLBC and associate with metastasis as well as poor prognosis; however, the mechanisms by which miR-221/222 function as oncomiRs remain unknown. Here, we report that miR-221/222 expression is inversely correlated with Notch3 expression in breast cancer cell lines. Notch3 is known to be overexpressed in luminal breast cancer cells and inhibits epithelial to mesenchymal transition (EMT). We demonstrate that miR-221/222 target Notch3 by binding to its 3′ untranslated region and suppressing protein translation. Ectopic expression of miR-221/222 significantly promotes EMT, whereas overexpression of Notch3 intracellular domain attenuates the oncogenic function of miR-221/222, suggesting that miR-221/222 exerts its oncogenic role by negatively regulating Notch3. Taken together, our results elucidated that miR-221/222 promote EMT via targeting Notch3 in breast cancer cell lines suggesting that miR-221/222 can serve as a potential therapeutic target in BLBC.

## MicroRNAs: how two non-coding RNAs promote tumor invasiveness

Two non-coding RNA molecules implicated in an aggressive subtype of breast cancer suppress the production of a key regulatory protein to enhance the migratory ability of breast cancer cells. Guo-Jun Zhang from Xiang’an Hospital of Xiamen University, China, and colleagues conducted a bioinformatics search for regulatory microRNAs that target the messenger RNA transcript encoding Notch3, a protein known to suppress tumor spread. Their analysis pinpointed microRNA-221 and microRNA-222, both previously implicated in the growth and spread of basal-like breast cancer. Experiments with cancer cell lines showed that the two microRNAs bind the tail end of Notch3’s transcript, which blocks protein production. Overexpressing the microRNAs made the cells more motile and invasive, whereas overexpression of Notch3 attenuated this process. The findings point toward microRNA-221 and microRNA-222 as potential drug targets for women with basal-like breast cancer.

## Introduction

The majority of breast cancer deaths result from metastatic disease.^1^ One of the pivotal processes that induce metastasis of cancers is the epithelial-to-mesenchymal transition (EMT) by which epithelial cells are converted to cells with a mesenchymal phenotype that is associated with enhanced migratory and invasive properties.^2^ EMT is considered to be the first step in the complex process of metastasis for many types of cancers.^2,3^ There have been conflicting views about the role of EMT in metastasis. Based upon the results from genetically engineered mouse models, some investigators found that EMT is not required for metastasis but has an important role in chemoresistance,^4,5^ while others have demonstrated that metastatic dissemination of mammary tumors indeed depends on EMT programs.^6^ In another example, pancreatic carcinoma cells have been shown to utilize EMT during metastatic dissemination.^7^

Recently, the discovery of microRNAs (miRNAs), which perform important regulatory functions in EMT, provides a novel strategy for the treatment of cancer invasion and metastasis. miRNAs are a class of small endogenous noncoding RNAs that are involved in regulating many biological processes by base-pairing with the 3′ untranslated region (UTR) of target messenger RNAs (mRNAs), resulting in their translational inhibition or degradation.^8^ Both miR-221 and miR-222 (miR-221/222), located on the X chromosome with the same seed sequences, are highly expressed during breast tumorigenesis and metastasis.^9,10^ MiR-221/222 are thought to serve as oncomiRs because they inhibit many tumor suppressors, including p27^KIP1^,^11^ FOXO3A,^12^ PTEN, and TIMP3.^13^ A mutual negative regulatory loop between miR-221/222 and ERα was also reported by De Leva et al.^12^ Moreover, miR-221/222 decrease E-cadherin expression by targeting the 3′-UTR of the GATA family-related TRPS1 (tricho-rhino-phalangeal syndrome type 1) and induces EMT by negative regulation of ZEB2.^14^ Overall, miR-221/222 have been shown to promote EMT, tumorigenesis, and metastasis through multiple mechanisms.

Notch family, including four Notch receptors (NOTCH1, NOTCH2, NOTCH3 and NOTCH4 (NOTCH1–4)) and five ligands of the Delta–Serrate–Lag (DSL) family (jagged 1 (JAG1), jagged 2 (JAG2), delta-like 1 (DLL1), delta-like 3 (DLL3) and delta-like 4 (DLL4)), plays vital roles in many biologic processes, including cell fate determination, stem cell maintenance, and lineage commitment.^15^ In human cancers, increasing evidence has demonstrated that the outcome of Notch activation is dependent on the cancer type and cellular context.^16–19^ It has been reported that Notch3 is specifically overexpressed in mouse epithelial cells and mammary luminal progenitor and is required for luminal breast filling by inhibiting apoptosis.^20,21^ Notch3 is elevated in luminal cells and gives rise to luminal lineages, restricting the proliferation and consequent clonal expansion of these cells.^22^ Interestingly, our previous study found that Notch3 is highly expressed in ER-positive luminal type compared with triple–negative breast cancers,^23,24^ demonstrating its opposite expression pattern to miR-221/222 in breast cancers. Furthermore, we also provided evidence for a pivotal role of Notch3 in the suppression of EMT and metastasis via trans-activating ERα in breast cancers.^23,24^

It is well-established that a single miRNA usually regulates a large set of target genes. It is likely that miR-221/222 target other genes that are involved in tumorigenesis and metastasis. In the current study, we demonstrated that Notch3 is a novel target of miR-221/222 which directly bind to its 3′UTR inhibiting its translation. We further validated that miR-221/222 suppress Notch3, ERα, and E-cadherin-induced EMT. These results indicate vital, multi-functional roles of miR-221/222 in the promotion of EMT in breast cancer.

## Results

### Notch3 is overexpressed in luminal breast cancer cells and has an inverse correlation with miR-221/222

**Fig. 1:**
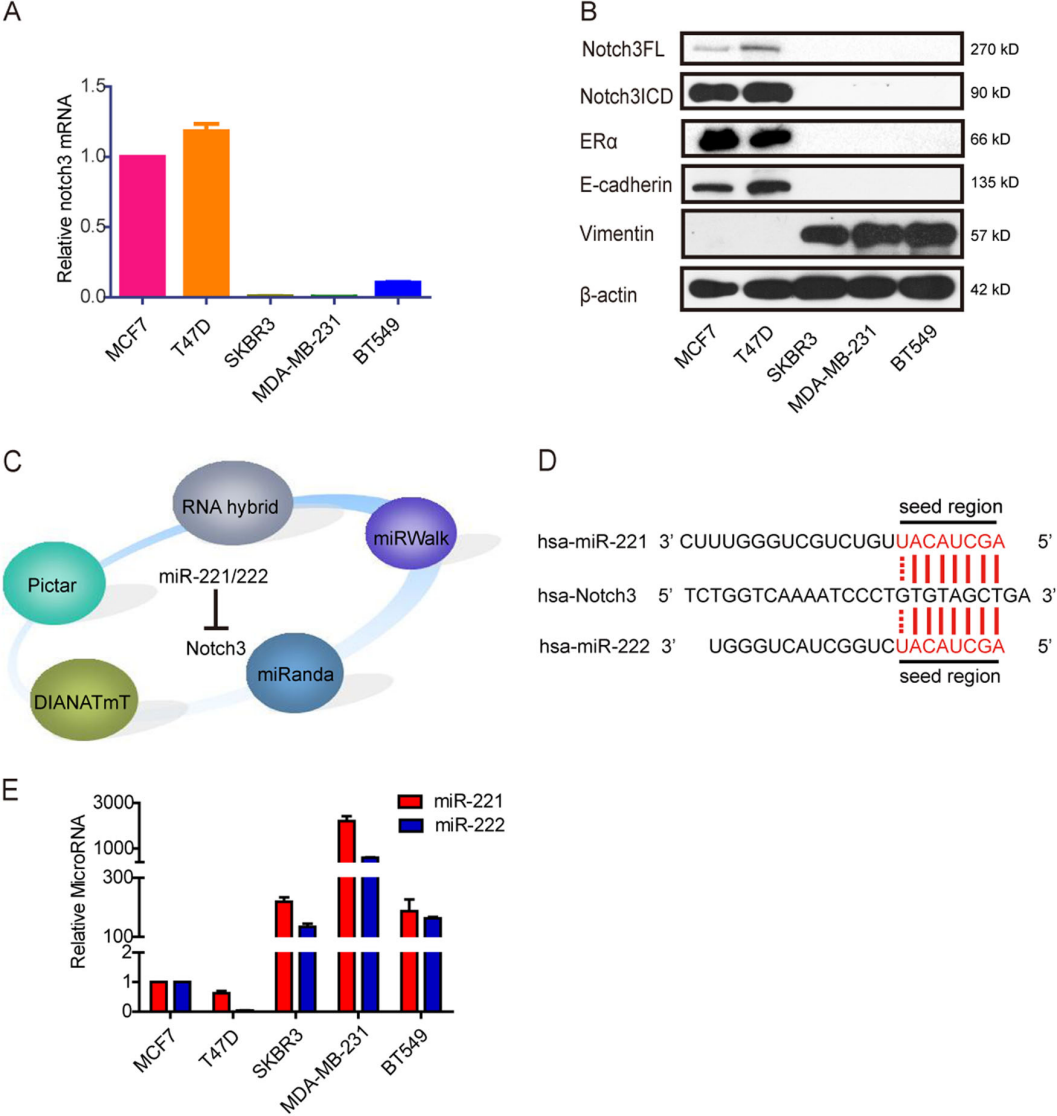
miR-221/222 have an inverse correlation with Notch3 in breast cancer cells. **a** qRT-PCR analysis of Notch3 mRNA expression in breast cancer cell lines. **b** Endogenous Notch3 full length (FL) and the intracellular domain (ICD), ERα, E-cadherin, and Vimentin protein levels in breast cancer cell lines. **c, d** Databases to predict miR-221/222 target genes and sequence alignment of the human miR-221 and miR-222 seed regions with Notch3 3′UTR, which contains predicted miR-221/222 binding site. **e** qRT-PCR analysis of miR-221/222 expression in breast cancer cell lines

Our earlier study has revealed that Notch3 maintained luminal phenotype and suppresses tumor metastasis in breast cancer. As shown in Fig. 1a, high levels of Notch3 mRNA were specifically expressed in the ERα-positive luminal breast cancer cell lines MCF-7 and T-47D, but not in the ERα-negative breast cancer cell lines MDA-MB-231, BT-549 and SK-BR-3., Western blotting showed that both full-length and intracellular domain (ICD) of Notch3 were mainly expressed in luminal epithelial phenotype MCF-7 and T-47D cell lines, as shown by increased ERα and E-cadherin expression (Fig. 1b). However, Notch3 expression was not detected in the basal-like breast cancer cell lines MDA-MB-231, BT-549, and SK-BR-3, which express the mesenchymal marker vimentin (Fig. 1b).

To investigate the mechanism of Notch3 down-regulation in basal-like breast cancer cell lines, we performed bioinformatic searches (including Pictar, DIANAmT, miRanda, miRWalk and RNAhybrid databases) to identify putative microRNAs targeting Notch3. Among the candidate microRNAs identified, the 3′-UTR of human Notch3 contains regions that match the seed sequences of hsamiR-221 and hsamiR-222 (Fig. 1c, d). Furthermore, we examined the endogenous levels of miR-221/222 by qRT- PCR. The expression of both miRNAs was almost undetectable in luminal MCF-7 and T47D breast cancer cells, but miR-221/222 were highly expressed in the basal-like subtypes SKBR3, MDA-MB-231, and BT549 cells (Fig. 1e). Since our data showed an inverse correlation between miR-221 or miR-222 and Notch3 expression in breast cancer cells, we speculated that Notch3 might be a target of miR-221/222.

### MiR-221/222 suppress Notch3 protein expression and induce EMT

**Fig. 2:**
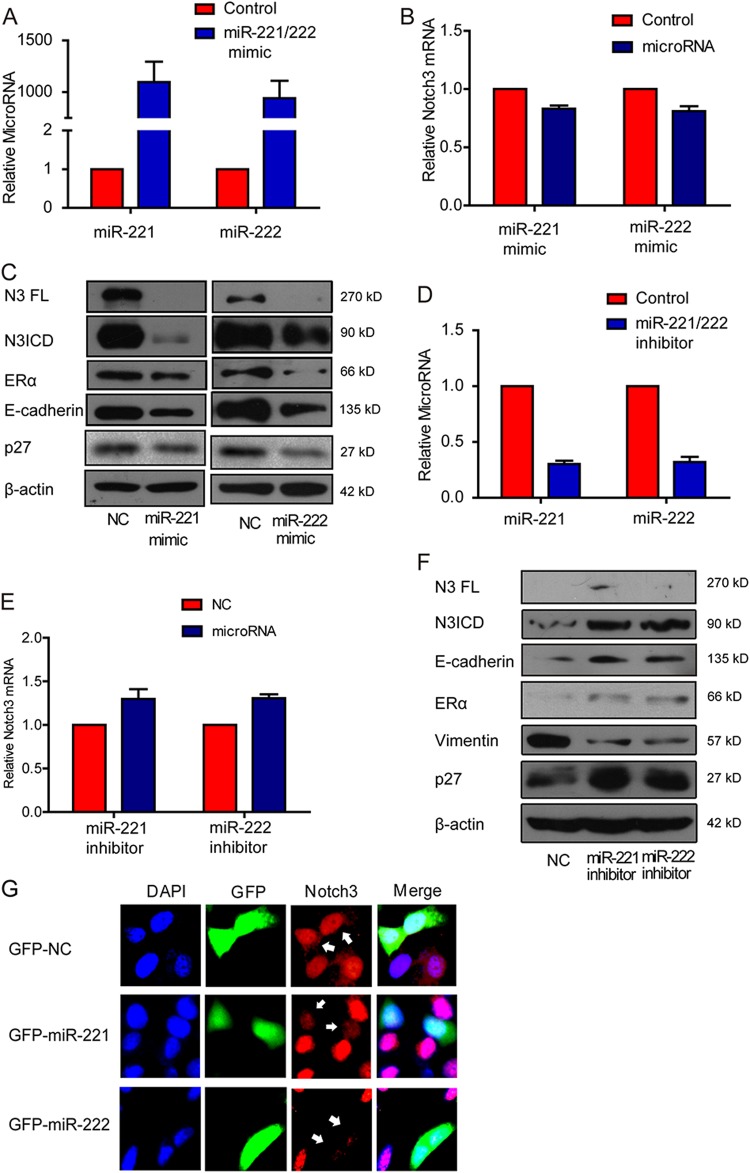
Notch3 is a target of miR-221/222 in breast cancer cells. **a** MiR-221 and miR-222 expression levels were detected by qRT-PCR after forced transfection of miR-221 or miR-222 mimics in MCF-7 cells. **b** qRT-PCR of Notch3 mRNA after forced transfection of miR-221 and miR-222 mimics in MCF-7 cells. **c** Western blot analysis of endogenous Notch3, ERα, p27 and E-cadherin protein levels in MCF-7 cells after ectopic expression of miR-221/222 mimics in MCF-7 cells. **d, e** qRT-PCR of endogenous miR-221/222 and Notch3 mRNA in MDA-MB-231 cells after transfection with miR-221/222 inhibitors. **f** Western blot analysis of N3FL, N3ICD, ERα, p27, and Vimentin protein levels after transfection with miR-221/222 inhibitors in MDA-MB-231 cells. **g** Immunofluorescence staining of MCF-7 cells transiently transfected with GFP vector (expressing only GFP), GFP-miR-221 or GFP-miR-222 using an anti-Notch3 antibody. Notch3 signals (red) in miR-221 and miR-222-transfected cells are labeled with arrowheads. DAPI, 4′,6-diamidino-2-phenylindole

To examine miR-221 and miR-222’s roles in regulating Notch3 and EMT, we transfected synthetic oligo mimics for miR-221 and miR-222 into MCF-7 breast cancer cells. Expression of both miRNAs was increased around 1000-fold after 48 h of transfection with the mimics (Fig. 2a). Using qRT-PCR, we found that Notch3 mRNA level did not change in miR-221 or miR-222 transfected cells compared with the control group (Fig. 2b). In contrast, overexpressing miR-221/222 significantly decreased endogenous Notch3 (both full length and ICD), ERα, p27, and E-cadherin protein levels (Fig. 2c). Conversely, the addition of miR-221/222 inhibitors to MDA-MB-231 cells (Fig. 2d) significantly induced Notch3 ICD and E-cadherin expression while vimentin expression significantly decreased. The p27, and ERα known to be miR221/222 targets, was also upregulated by miR-221/222 inhibition as expected (Fig. 2f). Intriguingly, Notch3 mRNA levels did not change in miR-221/222 inhibitor-transfected cells (Fig. 2e), indicating that miR-221/222 regulate Notch3 only at the translational level. To further confirm miR221/222’s role in regulating Notch3, we transiently transfected MCF-7 cells with pcDNA6.2-GW/EmGFP-miR-221/222 as well as the empty pcDNA6.2-GW/EmGFP vector. Immunofluorescence staining showed that Notch3 levels were largely reduced in cells expressing miR-221/222 compared with cells transfected with GFP alone or the untransfected cells (Fig. 2g).

### MiR-221 and miR-222 directly target Notch3 3′UTR to inhibit Notch3 expression

**Fig. 3:**
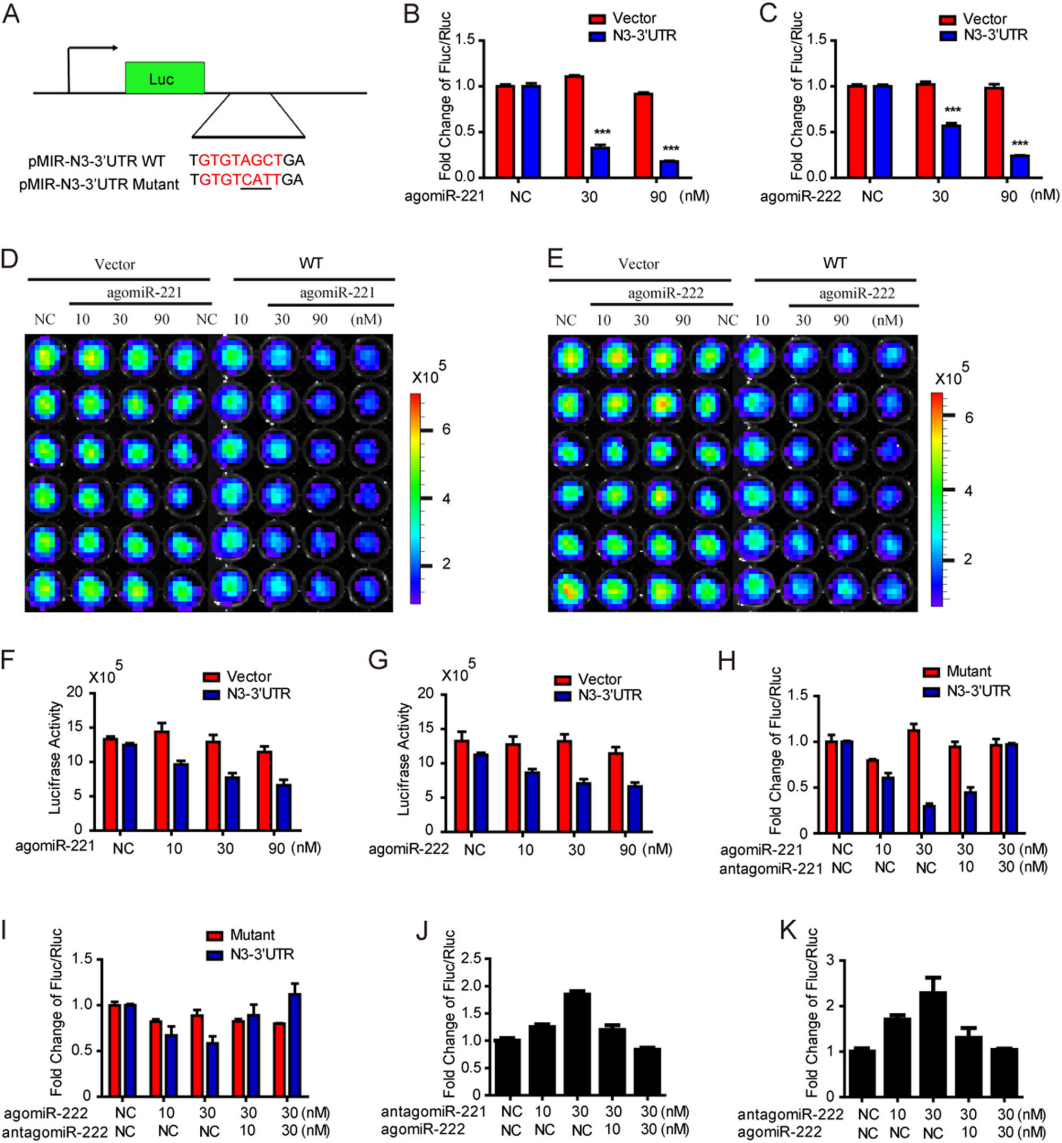
MiR-221/222 directly target Notch3 3′UTR. **a** The schematic luciferase reporter containing predicted Notch3 wild-type (WT) or its mutant 3′UTR sequences for miR-221/222. **b, c** MCF-7 cells were co-transfected with pMIR-Notch3-3′UTR WT or pMIR control reporters in the presence of agomiR-221/222 (10, 30, and 90 nM) or miR-control (NC). Fluc/Rluc activities were measured 48 h post-transfection and normalized to the corresponding vector control. Each transfection was performed in triplicate, and the experiment was repeated three times. Data represent the mean ± SEM. ***P *<* 0.001. **d, e** MCF-7 cells stably transfected with pMIR-Notch3-3′UTR were treated with agomiR-221/222 at the indicated concentrations or pMIR-control vector, and luciferase activities were detected 48 h after transfection by a molecular imaging device (IVIS KINETIC). **f, g** Quantitation of luciferase activities when transfected with agomiR-221 and agomiR-222 in IVIS KINETIC system. **h, i** MCF-7 cells were co-transfected with pMIR-Notch3-3′UTR wild-type (WT) or pMIR-Notch3-3′UTRmutant (Mutant) reporters in the presence of agomiR-221/222 (10, 30 nM) and antagomiR-221/222 (10, 30 nM) or miR-control (NC). Fluc/Rluc activities were measured 48 h post-transfection and normalized to the corresponding vector control. **j, k** MDA-MB-231 cells were co-transfected with pMIR-Notch3-3′UTR reporters in the presence of antagomiR-221/222 (10, 30 nM) and agomiR-221/222 (10, 30 nM) or miR-control (NC). Fluc/Rluc activities were measured 48 h post-transfection and normalized to the corresponding vector control. Each transfection was performed in triplicate, and the experiment was repeated three times. Data represent mean ± SEM

To verify that Notch3 is a direct target of miR-221/222, the wild type (WT) Notch3 reporter pMIR-Notch3-3′UTR WT or control reporter was co-transfected with agomiR221/222 (mimics) in MCF-7 cells that express low levels of miR-221 and miR-222 (Fig. 3a). As expected, both agomiR-221 and agomiR-222 decreased luciferase activities of the Notch3 reporter by about 50~80% in MCF-7 cells in a dose-dependent manner, while the control reporter was not affected (Fig. 3b, c). Similarly, luciferase activities were also reduced by agomiR-221/222 as visualized by bioluminescent imaging and following quantification (Fig. 3d–g). To further examine whether miR-221/222 directly regulates Notch3 by targeting the GTGTAGCT seed binding site, we co-transfected pMIR-Notch3 3′UTR WT or mutated reporters with miR-221/222 mimics and inhibitors (antagomiR-221/222) at the indicated concentrations in MCF-7 cells. Interestingly, the dose-dependent inhibitory effect of miR-221/222 on Notch3 3′UTR activity was rescued by co-expression of miR-221/222 inhibitors. However, no significant changes in luciferase activity were seen when using the Notch3 3′UTR mutant reporter (Fig. 3h, i). In contrast, miR-221/222 inhibitors increased luciferase activity in MDA-MB-231 cells in a dose-dependent manner when co-transfected with a pMIR-Notch3-3′UTR WT reporter. Increased luciferase activity was reversed when co-expressed with miR-221/222 mimics (Fig. 3j, k). In summary, these results support the bioinformatic prediction that Notch3 is a direct target of miR-221 and miR-222.

### MiR-221/222 induce EMT through downregulation of Notch3

**Fig. 4:**
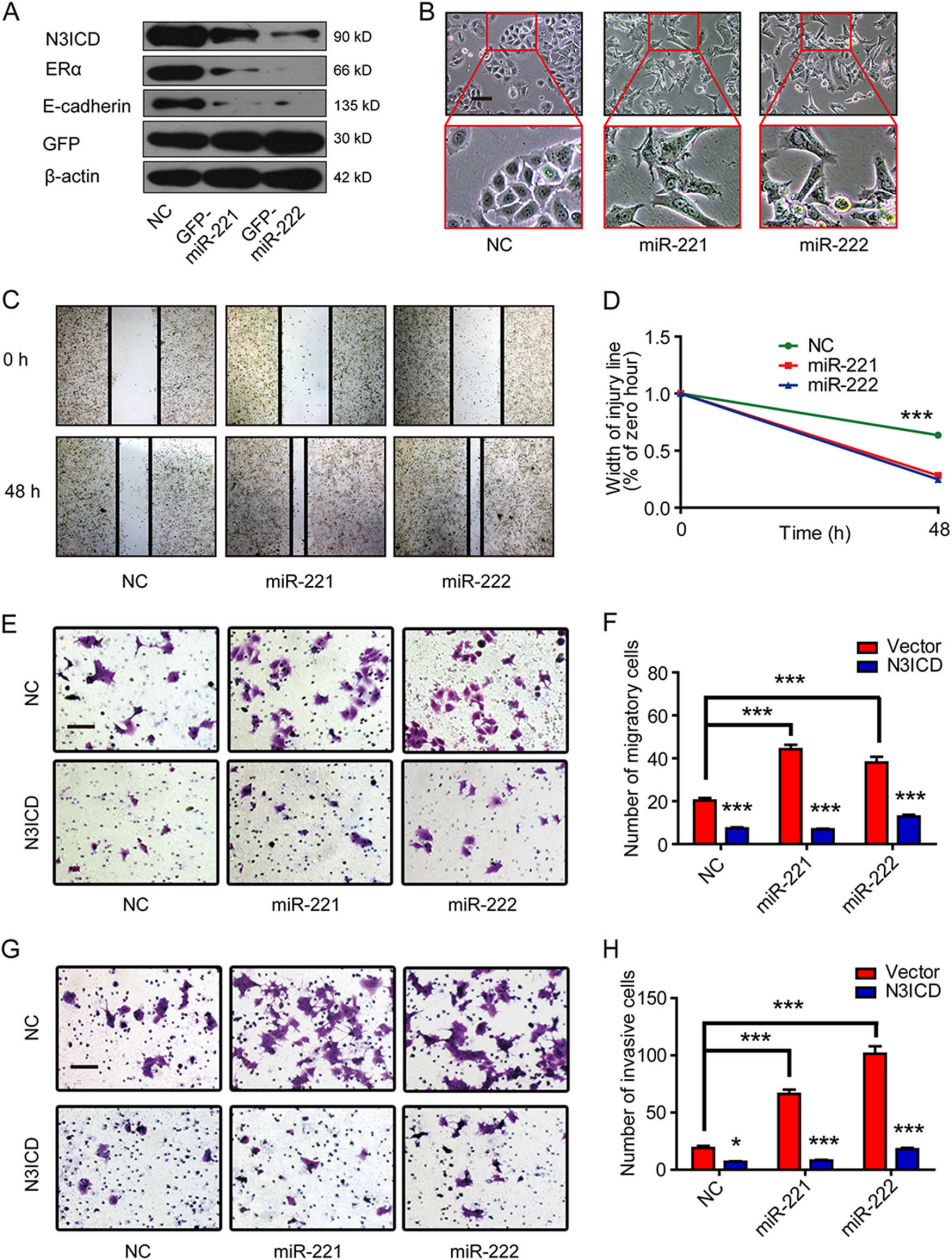
N3ICD overexpression reverses EMT promotion by miR-221 and miR-222 in MCF-7 cells. **a** Western blot shows that MCF-7 cells that were stably transfected with GFP-miR-221 and GFP-miR-222 vectors suppressed Notch3 ICD and ERα as well as E-cadherin. **b** Effect of miR-221/222 overexpression on cell morphology evaluated by phase contrast microscopy (×100). Scale bars represent 50 μm. **c, d** Representative micrographs (×40) of the wound healing assay in MCF-7 cells. Cells were photographed to measure wound length at 0 and 48 h. Wound healing lengths were measured in 5 random fields. Data represent the mean of triplicate experiments ± SEM. **e–h** Representative micrographs (×200) of migration and invasion Transwell assays. Stably transfected miR-221/222 MCF-7 cells were co-transfected with N3ICD or a control plasmid. Invading or migrating cells were counted in 5 random fields. Data represent the mean of triplicate experiments ± SEM. Scale bars represent 50 μm

**Fig. 5:**
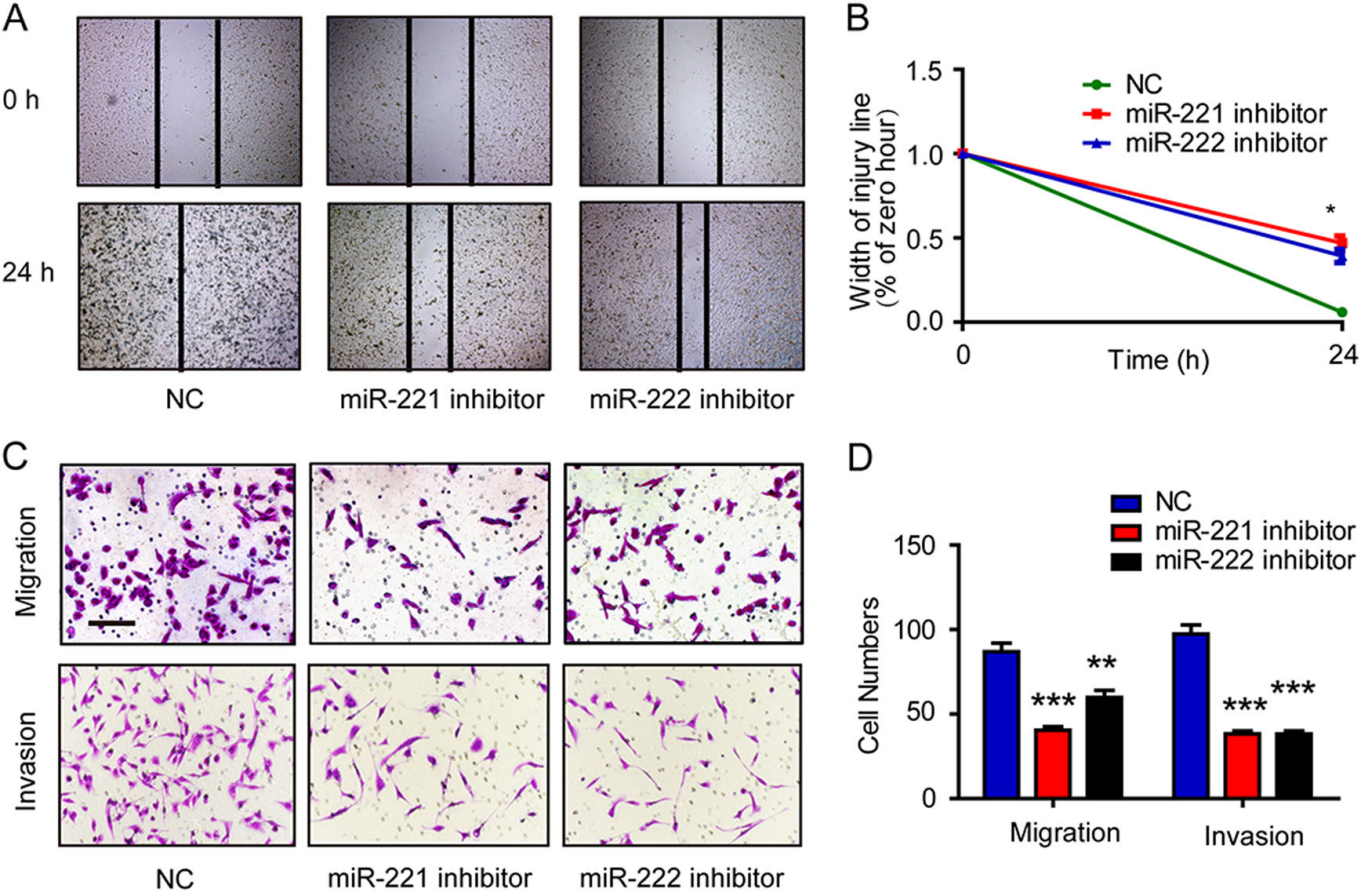
Inhibition of miR-221/222 decreases migration and invasion ability in MDA-MB-231 breast cancer cells. **a, b** Representative panels from the scratch wound assay in MDA-MB-231 cells imaged by microscopy. MDA-MB-231 cells were transfected with a miR-221/222 inhibitor or miR-control. Wound healing was measured in 5 random fields. Data represent the mean of triplicate experiments ± SEM. **c, d** Representative micrographs (×200) of matrigel-coated or non-coated Transwell assays in MDA-MB-231 cells transfected with miR-221/222 inhibitors or miR-NC. Cells were photographed to measure wound length at 0 and 24 h. Invading or migrating cells were counted in five random fields. Data represent the mean of triplicate experiments ± SEM. Scale bars represent 50 μm. *P *<* 0.05; **P *<* 0.01; ***P *<* 0.001

To determine the effects of miR-221 and miR-222 on EMT, we established MCF-7 cells stably transfected with GFP-miR-221 or GFP-miR-222 plasmids. Western blot analysis revealed that overexpression of miR-221 or miR-222 downregulated Notch3, ERα, and E-cadherin expression in MCF-7 cells (Fig. 4a). Subsequently, we examined whether overexpression of miR-221/222 induced morphological changes associated with EMTs in MCF-7 cells. Both miR-221- and miR-222-transfected MCF-7 cells displayed fibroblast-like morphologies and showed cellular scattering compared with the control cells, which maintained a cobblestone phenotype with strong cell-cell adhesion (Fig. 4b). Next, we investigated whether miR-221 and miR-222 affect cellular migration and invasion in breast cancer cells. It had been reported that miR-221/222 promote proliferation in human cancers. To eliminate these impacts, serum free medium was used in wound healing assay and transwell assay (Supplementary Figure 1A–D). In a wound healing assay, miR-221 or miR-222 overexpression in MCF-7 cells resulted in an approximately 2-fold increase in the cell migration rate compared with the control group after 48 h in culture (Fig. 4c, d and Supplementary Figure 1E). Moreover, matrigel-coated (for invasion) or uncoated (for migration) Transwell assays showed that miR-221 and miR-222 overexpression drastically increased the migration (about 2-folds) and invasiveness (5-folds) of the MCF-7 cell line; when overexpressing N3ICD by transient transfection, the increases in invasion and migration were almost completely suppressed (Fig. 4e–h). Conversely, expressing miR-221 or miR-222 inhibitors in MDA-MB-231 cells decreased the migration rate by about 50% compared with the control group after 24 h (Fig. 5a, b). MDA-MB-231 cells treated with either miR-221 or miR-222 inhibitor showed significantly decreased migratory and invasive abilities compared to control cells (Fig. 5c, d). These results confirm that miR-221 and miR-222 promote migration and invasion through inhibition of Notch3 expression.

## Discussion

MicroRNAs are attractive and effective drug targets as they regulate many important cellular processes and are differentially expressed in malignancies compared with normal tissues. Numerous miRNAs have been shown to have altered expression in a variety of human cancers and function as oncomiRs or tumor suppressors.^25–27^ For example, constitutive up-regulation of miR-221/222 contributes to poor prognosis in various types of tumors, including hepatocarcinoma, colon, breast, pancreas and lung cancers.^12,13,28,29^ In breast cancer, miR-221/222 was more abundant in basal-like tumors than in ER/PR–positive tumors^14^ which in line with our previous findings. It has been demonstrated that miR-221 and miR-222 each directly target ERα mRNA at a conserved site and are involved in anti-estrogen resistance.^12,30–32^ Our previous study also revealed that Notch3 trans-activated ERα and inhibited EMT and metastasis in breast cancer.^23^

In the present study, we attempted to identify potential targets regulated by miR-221/222. Consistent with our previous findings, elevated expression of miR-221 and/or miR-222 in basal-like/mesenchymal/ER-negative breast cancer cells compared to luminal/epithelial/ERα-positive cells was detected by RT-PCR.^12^ Notch3 was elevated in ER-positive breast cancer cells, indicating that miR-221/222 could be a causal factor in the downregulation of Notch3. This notion was further confirmed by the observation that Notch3 was significantly inhibited in ERα-positive breast cancer cells treated with miR-221/222 mimics in a dose-dependent manner. Conversely, overexpression of miR-221/222 inhibitors upregulated Notch3 expression and downstream proteins, such as ERα and E-cadherin. Also, the fact that Notch3 mRNA was not affected by either mimics or inhibitors suggests that miR-221/222 directly binds to Notch3 transcripts without leading to its degradation. This finding is consistent with a previous report showing that miR-221/222 can suppress the expression of targeted proteins without altering their mRNAs.^13^ Hence, we do believe that the low Notch3 mRNA and protein expressions in basal-like breast cancer cells are not just because of the abundant of miR-221/222 but a complex regulatory network.

MiR-221/222 have been shown to increase migration and invasion by targeting PTEN and p27 indicating their potential mechanistic link to EMT.^11,13^ MiR-221/222 also target the 3′UTR of various genes, including those encoding estrogen receptors (ESR1) and TRPS1. It had been reported that miR-221/222 could directly bind to the ERα-3′UTR and suppress its expression, on the other hand, ERα also could directly represses miR-221 and -222 by recruiting the corepressors NCoR and SMRT.^12^ In addition, forced expression of ERα repressed Slug, up-regulated E-cadherin, and induced cells to grow as adherent colonies with reduced invasiveness.^33^ What’s more, over-expression of miR-221/222 decrease E-cadherin through targeting the 3′UTR of the GATA family-related TRPS1, which represses ZEB2 expression.^14^ Thus, there seem a complex regulatory network between miR-221/222, ERα, and E-cadherin. Therefore, it’s not clear if miR-221/222 regulated ER and E-cadherin independently or not. At least, miR-221/222 could regulate E-cadherin either independent or dependent on ERα regulation. To investigate the functional significance of Notch3/ERα regulation by miR-221/222, we performed the migration, invasion, and wound healing assays using mimics or inhibitors against miR-221 and/or miR-222. As expected, addition of inhibitors to mimics or addition of mimics to inhibitors reversed their biological functional effects on migration, invasion, and wound healing. The observation that the overexpression of miR-221/222 induces migration and invasion provides functional evidence for the possible role of these miRNAs in breast cancer. The invasive potential of miR-221/222 was strongly blocked by the ectopic expression of Notch3 ICD, indicating that miR-221/222 promote EMT, at least partially, through targeting Notch3.

Our findings have uncovered the regulatory network of miR221/222 and the underlying mechanism for their functional roles as oncomiRs in breast cancer cell lines. The data presented in this study highlight a mechanism by which miR-221/222 promote migration and invasion by targeting Notch3 in breast cancer cell lines. Our results suggest that targeting miR-221 and miR-222 represents an effective strategy to prevent and inhibit tumor progression and metastasis in breast cancer.

## Materials and methods

### Cell culture and transfection

MCF-7, T47D, SKBR3, BT549, and MDA-MB-231 cell lines were purchased from and validated by American Type Culture Collection (ATCC, VA, USA) and cultured in DMEM medium, supplemented with 10% FCS. All cells were cultured at 37 °C in a humidified 5% CO_2_ atmosphere. The oligonucleotides sequences of the miR-221/222 inhibitors, miR-221/222 mimics, agomiR-221/222, antagomiR-221/222 (displayed in Supplementary Table S1) were transfected by Lipo2000. The plasmids of pcDNA6.2-GW/EmGFP-miR-221/222 were obtained from Professor Cheng^32^ (H. Lee Moffitt Cancer Center and Research Institute). The plasmids of pCLE-N3ICD (#26894) and control vector pCLE (#17703) were purchased from Addgene (MA, USA). The vectors containing these oligonucleotides sequences were transfected into MCF7 cell line by using Lipo2000. Ten μg/ml Blasticidin S was added to the medium after 2 days in culture for selection.

### RNA purification and real-time PCR analysis

Total RNA containing small RNAs was extracted from cancer cells using Trizol total RNA isolation reagent (Invitrogen, CA, USA) according to the manufacturer’s instructions. cDNA was synthesized from total RNA using PrimeScript RT reagent Kit (Takara, Japan) according to the protocol. Quantitative real-time PCR was performed with a SYBR Select Master Mix (Thermo Fisher, MA, USA) and the CFX96 Real-time PCR Detection System (Bio-Rad, CA, USA). MicroRNA real-time transcription-PCR quantification of miRNA expression was carried out using a Hairpin-it miRNAs RT-PCR Quantitation Kit and TaqMan-microRNA assay kit (GenePharma, Suzhou, China) according to the manufacturer’s protocol. U6 was used for normalization. Primer sequences are listed in the Supplementary Table S1.

### Western blot analysis

Protein extraction and Western blotting were performed as described previously.^34^ Briefly, cells were harvested in RIPA lysis buffer (Millipore, USA). Protein lysates were electrophoresed in polyacrylamide gels and transferred to PVDF membranes. After blocking in 5% skim milk, the membranes were incubated with primary antibodies overnight at 4 °C. The secondary antibody was diluted 1:3000 in fresh blocking solution. The blots were visualized using the X-OMAT film (Kodak, Japan). All blots were derived from the same experiment and processed in parallel. Primary antibodies are listed in Supplementary Table S2.

### Wound healing assay

Cells were starved in serum-free medium for 24 h before injury lines were applied. The injury line was made with a tip 2 mm wide on cells plated in culture dishes at 90% confluency. After rinsing with phosphate-buffered saline, cells were allowed to migrate in serum-free medium. Photographs were taken (×40) after 24 h (MDA-MB-231) or 48 h (MCF-7). An average of five random widths of injury lines were measured for quantitation.

### Transwell assay

Cells were serum-starved for 24 h before seeding in Matrigel-coated/uncoated chambers (8μm pore size, BD Bioscience, USA). 5 × 10^4^ MCF-7 cells or 2 × 10^4^ MDA-MB-231 cells were seeded in the upper chamber with serum-free medium. Complete medium was added to the bottom chamber. After 48 h cell culturing, cells were stained with 0.1% crystal violet. Each assay was performed in triplicate. Cells from five fields in each well were counted by two investigators.

### Dual-luciferase reporter assays

Luciferase activities were detected by using dual luciferase reporter assay kit (Promega, WI, USA). The 3′UTR or mutated 3′UTR of the Notch3 gene was cloned between SpeI/HindIII sites of the luciferase reporter vector pMIR (pMIR-Notch3-3′UTR was kindly provided by Prof. Paola Zanovello, University of Padova^35^). pRL-SV40 (Promega, WI, USA) was used as the control vector to balance transfection efficiency. To detect the regulation of Notch3 mRNA by miR221/222, pMIR-Notch3-3′UTR-wt or pMIR-Notch3-3′UTR-mut were co-transfected into MCF-7 or MDA-MB-231cells with different doses of miR221 mimics and/ or miR221 inhibitors.

### Statistical analysis

Data were statistically analyzed using two-sided Student’s *t*-test. *P* *<* 0.05 was considered statistically significant. Data are shown as mean ± s.e.m. unless otherwise stated.

### Data availability

All data supporting our findings can be found in the main paper or in supplementary files.

## Electronic supplementary material

Supplementary

## Electronic supplementary material

**Supplementary information** accompanies the paper on the *npj Breast Cancer* website (10.1038/s41523-018-0073-7).
