# Supplementary material for: MiR-221/222 promote epithelial-mesenchymal transition by targeting Notch3 in breast cancer cell lines
Source: NPJ Breast Cancer. 2018 Aug 6;4:20. doi: 10.1038/s41523-018-0073-7 (PMC6079079; doi:10.1038/s41523-018-0073-7)

Table S1. Oligonucleotide sequences

Assay		Sequences (5' to 3')		Amplicon (bp)
RT-PCR				
	Notch3	F	ATGCAGGATAGCAAGGAGGA	86
		R	AAGTGGTCCAACAGCAGCTT	
	ERα	F	CTCTCCCACATCAGGCACA	157
		R	CTTTGGTCCGTCTCCTCCA	
	E-cadherin	F	AAAGGCCCATTTCCTAAAAACCT	172
		R	TGCGTTCTCTATCCAGAGGCT	
	Vimentin	F	GACGCCATCAACACCGAGTT	238
		R	CTTTGTCGTTGGTTAGCTGGT	
	β-actin	F	GAGACCTTCAACACCCCAGCC	264
		R	AATGTCACGCACGATTTCCC	
microRNA				
	miR-221 mimic	F	AGCUACAUUGUCUGCUGGGUUUC	
		R	AACCCAGCAGACAAUGUAGCUUU	
	miR-222 mimic	F	AGCUACAUCUGGCUACUGGGU	
		R	CCAGUAGCCAGAUGUAGCUUU	
	agomiR-221	F	AGCUACAUUGUCUGCUGGGUUUC	
		R	AACCCAGCAGACAAUGUAGCUUU	
	agomiR-222	F	AGCUACAUCUGGCUACUGGGU	
		R	CCAGUAGCCAGAUGUAGCUUU	
	miR-NC	F	UUCUCCGAACGUGUCACGUTT	
		R	ACGUGACACGUUCGGAGAATT	
	miR-221 inhibitor	CAAACCCAGCAGACAAUGUAGCU		
	miR-222 inhibitor	ACCCAGUAGCCAGAUGUAGCU		
	antagomiR-221	CAAACCCAGCAGACAAUGUAGCU		
	antagomiR-222	ACCCAGUAGCCAGAUGUAGCU		

Table S2. Description of antibodies

**Antibodies**	**Vendor**	**Catalog number**	**NO.**
Notch3	CST	2	5276
ERα	CST	4	8644
E-cadherin	CST	13	3195
Vimentin	CST	1	5741
P27	CST	4	3686
β-actin	Santa Cruz	C3012	Sc-47778
ERα	Santa Cruz	12613	Sc-8002
Notch3	Santa Cruz	12013	Sc-5593

Supplementary Figure 1

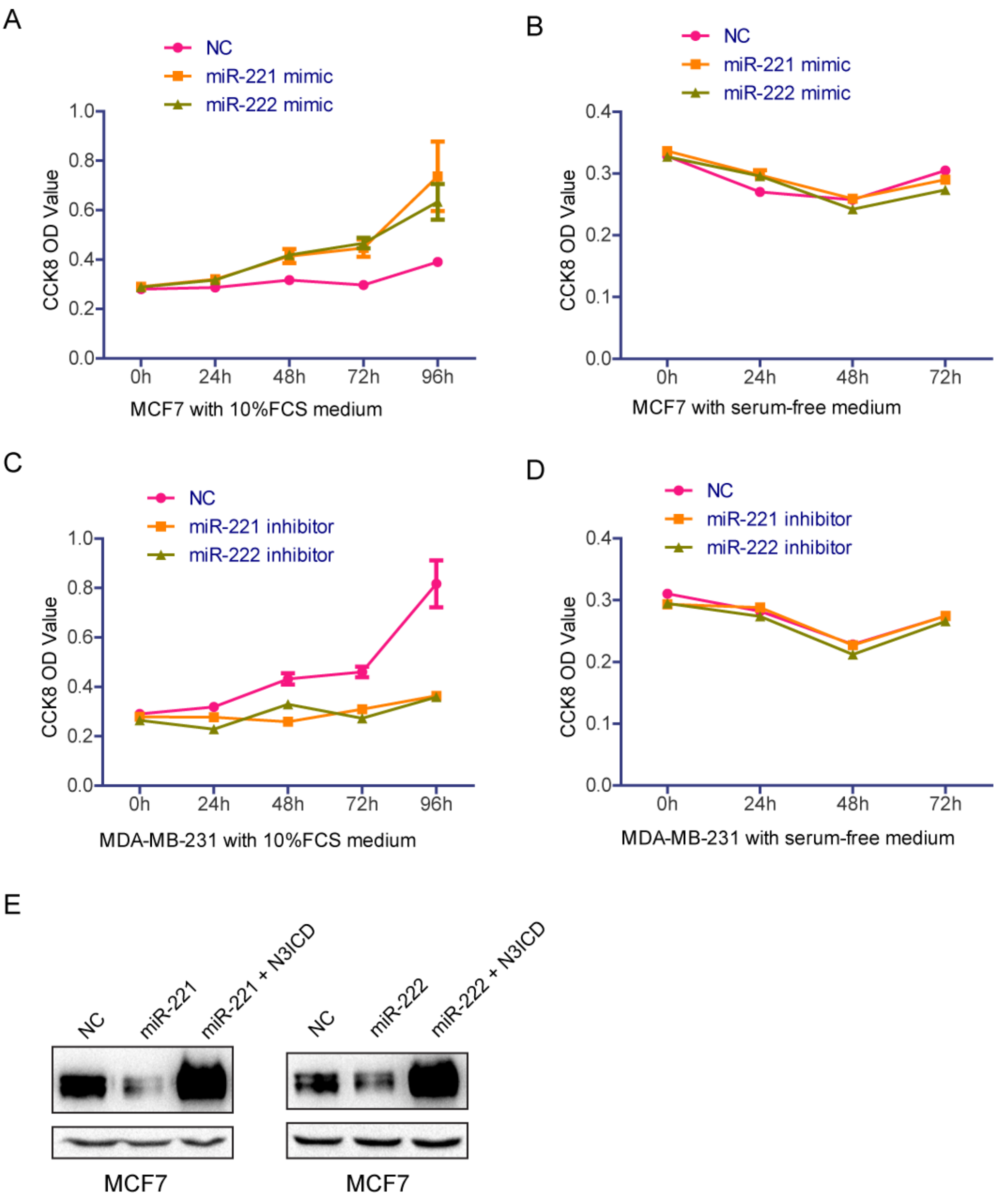

Supplementary Figure 1 (A) CCK8 assay showing that MCF7 cell transfected with miR-221 or miR-222 mimic proliferated a higher rate than control group in 10% FCS medium. **(B)** CCK8 assay shows the proliferate rate of MCF7 cell transfected with miR-221 or miR-222 mimic compared with control group in serum-free medium. **(C)** CCK8 assay showing that MDA-MB-231 cell transfected with miR-221 or miR-222 inhibitor proliferated a higher rate than control group in 10% FCS medium. **(D)** CCK8 assay shows the proliferate rate of MDA-MB-231 cell transfected with miR-221 or miR-222 inhibitor compared with control group in serum-free medium. **(E)** Western blot analysis of expression of Notch3ICD in

MCF-7 cells after ectopic expression of miR-221/222 and pCMV-N3ICD in MCF-7 cells.

Un-processed WB blots

Figure 1

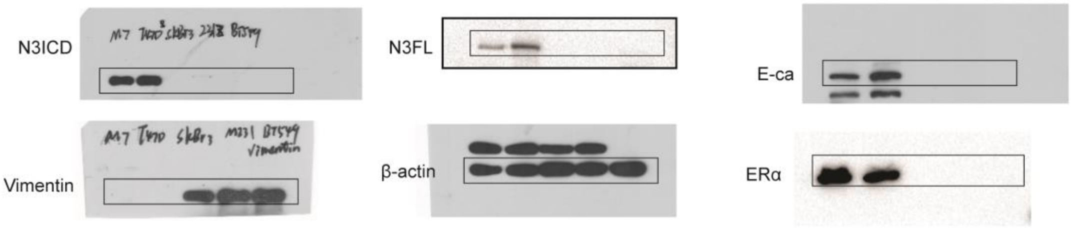

Figure 2

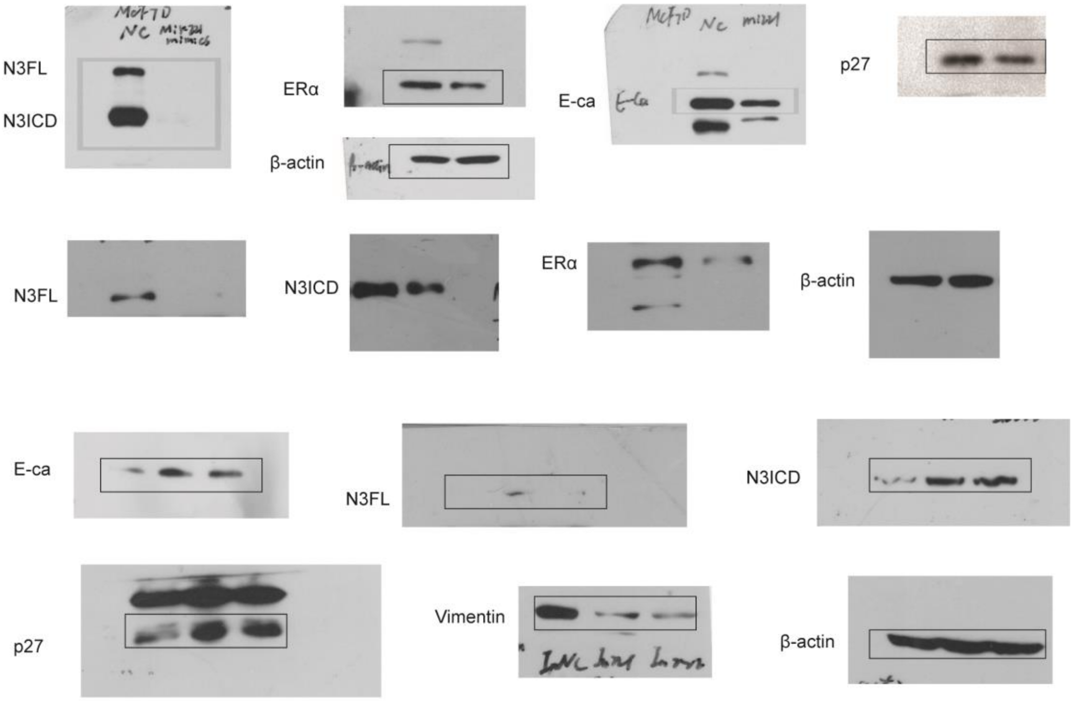

Figure 4

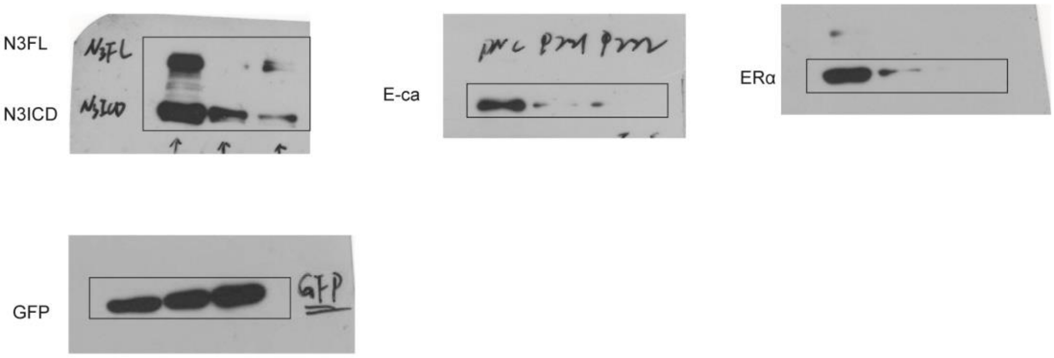

Supplement: Supplementary file 1 — Supplementary [file 41523_2018_73_MOESM1_ESM.pdf]
